# Strengthening hepatitis B and C surveillance in Europe: results from the two global hepatitis policy surveys (2013 and 2014)

**DOI:** 10.1186/s41124-016-0009-5

**Published:** 2016-06-30

**Authors:** Jeffrey V. Lazarus, Antons Mozalevskis, Kelly Safreed-Harmon, Irina Eramova

**Affiliations:** 1 CHIP, Centre for Health and Infectious Disease Research and WHO Collaborating Centre on HIV and Viral Hepatitis, Rigshospitalet, University of Copenhagen, Copenhagen, Denmark; 2 World Health Organization (WHO) Regional Office for Europe, Copenhagen, Denmark

**Keywords:** Hepatitis, Surveillance, Survey, Europe

## Abstract

**Background:**

Hepatitis B and C are major public health threats in the World Health Organization (WHO) European Region. Viral hepatitis surveillance shortcomings have resulted in many WHO Member States having insufficient data available to guide decision-making. This study describes surveillance in the region based on a quantitative sub-analysis of findings from the 2013 WHO viral hepatitis policy report and a qualitative analysis of civil society survey responses associated with these findings.

**Methods:**

Descriptive statistics were created from information that national government focal points for viral hepatitis in 44 countries had previously reported in response to the WHO survey. Bivariate analysis was performed to compare data from within and outside of the European Union/European Economic Area (EU/EEA). Survey responses from civil society organizations in the countries of the WHO European Region were collated, and a descriptive analysis of the comments on surveillance-related questions was performed to identify key themes.

**Results:**

The response rate for the survey of governments was 83 % among both EU/EEA countries (25/30) and non-EU/EEA countries (19/23). More than 90 % of governments reported having national surveillance systems for the acute forms of hepatitis B and hepatitis C, but less than two-thirds reported surveillance for the chronic forms of both diseases. High proportions of governments reported having central registries for the reporting of deaths (96 %) and liver cancer cases (80 %), while less than half reported regularly conducting viral hepatitis sero-surveys. All responding Member States reported having adequate laboratory capacity nationally to support hepatitis outbreak investigations and other surveillance activities. Target populations for sero-surveys most commonly included people who inject drugs (27 %), the general population (25 %), men who have sex with men (20 %) and pregnant women (20 %). Few statistically significant differences were found between EU/EEA and non-EU/EEA countries.

**Conclusions:**

Study findings indicated a capacity for robust viral hepatitis surveillance across the WHO European Region, with most countries having important surveillance components in place, but notable weaknesses were also identified. There is an urgent need for countries throughout the region to strengthen their surveillance programs in order to maximize the population-level impact of advances in HBV and HCV prevention and treatment.

**Electronic supplementary material:**

The online version of this article (doi:10.1186/s41124-016-0009-5) contains supplementary material, which is available to authorized users.

## Background

Hepatitis B virus (HBV) and hepatitis C virus (HCV) are increasingly recognized as major public health threats worldwide. These two viruses are estimated to cause approximately 1.4 million deaths annually [1]. An estimated 57 % of liver cirrhosis cases and 78 % of primary liver cancer cases are attributable to HBV and HCV infections [2]. In the World Health Organization (WHO) European Region, more than 18 million adults are estimated to be carrying the hepatitis B surface antigen (HBsAg) [3], which is a marker of chronic HBV infection. An estimated 15 million adults in this region have HCV-RNA [3], which is a marker of HCV infection. Two-thirds of HBsAg-positive people and HCV-RNA-positive people live in the countries outside of the European Union (EU) and European Free Trade Association (EFTA), even though those countries account for only 42 % of the WHO European Region’s adult population [3]. Throughout the Region, high levels of HCV transmission, and to a lesser extent, HBV transmission, are known to occur through injecting drug use [4]. Eighty percent of new HCV infections with a known transmission route in EU and European Economic Association (EEA) countries are attributable to injecting drug use [5]. Regarding HBV, additional transmission pathways of note in the region are heterosexual sex and sex between men. In some European countries, nosocomial transmission of HBV also contributes to the disease burden. Available epidemiological evidence on HBV and HCV among migrants suggests that many migrant groups are also disproportionately affected in several European countries [3, 6, 7].

Major advances in the prevention and treatment of chronic HBV and HCV infections are influencing the course of both epidemics. An effective hepatitis B vaccine became available in 1982, and the mass immunization campaigns that followed led to a sharp decline in new infections. For example, a recent study in Tajikistan demonstrated seven-fold lower prevalence of HBsAg among the vaccinated birth cohorts compared with the baseline among unvaccinated cohorts [8]. The strategy of providing the first dose of HBV vaccine within 24 h of birth is effective for preventing mother-to-child HBV transmission and may ultimately help to bring about the eradication of the virus [9]. In the last decade, treatment options for chronic HBV and HCV infection have dramatically improved, resulting in better short-term and long-term survival as well as better quality of life for people with these diseases. Effective antiviral agents can supress replication of the hepatitis B virus in most infected people [10], and recent HCV treatment breakthroughs have made it possible to cure chronic HCV infection in more than 90 % of patients [11]. Consequently, people with access to HBV and HCV treatment can greatly reduce their risk of developing cirrhosis, liver cancer and liver failure [10, 12]. In light of the potential impact of these developments, combined with other essential prevention interventions, WHO has made the elimination of viral hepatitis as a public health threat by 2030 the overarching goal of its ambitious first-ever global health sector strategy on viral hepatitis [13].

In order to take full advantage of advances in viral hepatitis prevention, treatment and care, decision-makers need to work from a strong evidence base that includes accurate surveillance data [14, 15]. Case reporting, based on regular notification by clinicians and laboratories, has traditionally been the foundation of viral hepatitis surveillance. At the same time, other information sources aimed at assessing disease burden and outcomes, such as serological surveys and cancer and death registries, are important for measuring the impact of hepatitis infections and evaluating the efficacy of interventions [14]. The importance of reliable information gathered through epidemiological surveillance was recognized by the World Health Assembly in its 2010 resolution on viral hepatitis [16]. Four years later, in a second viral hepatitis resolution, the World Health Assembly noted that “most Member States lack adequate surveillance systems for viral hepatitis to enable them to take evidence-based policy decisions” and repeated its 2010 call for Member States (MS) to remedy this situation [17]. The new resolution also called on MS to develop and implement national viral hepatitis strategies “based on the local epidemiological context,” further calling attention to the need for robust surveillance. WHO has provided technical support to MS by publishing in February 2016 its first comprehensive viral hepatitis surveillance guidance [18] and in April 2016, a monitoring and evaluation framework for the global health sector strategy on viral hepatitis [19].

In recent years, the European Centre for Disease Prevention and Control (ECDC) has led efforts to improve and harmonize viral hepatitis surveillance in the 28 EU MS, along with three EEA countries (Iceland, Liechtenstein and Norway). A key ECDC accomplishment has been the introduction of an enhanced hepatitis B and C surveillance program, which calls for EU/EEA countries to provide case-based data using standardized case definitions to the European Surveillance System (TESSy), a web-based data management platform [20]. Two 2015 publications indicated that, while the program has brought about considerable improvements to the quality of the data collected, some aspects of viral hepatitis surveillance, such as the completeness of the data and the task of classifying cases as acute or chronic, remain challenging. Furthermore, the current EU case definitions have yet to be adopted in all countries, which makes it difficult to compare data among countries [21, 22].

No comparable supra-national initiative has been undertaken in the non-EU/EEA countries. Publications documenting viral hepatitis surveillance practices in non-EU/EEA countries are scarce. The purpose of this study is to describe HBV and HCV surveillance policies and practices across the entire WHO European region based on a sub-analysis of surveillance-related findings from the WHO global viral hepatitis policy survey [23] and to present complementary qualitative findings from a civil society survey [24].

## Methods

We carried out the following study using two existing datasets.

### WHO global viral hepatitis policy survey

WHO, in collaboration with the World Hepatitis Alliance and the University of Copenhagen, conducted a survey of national policy responses to viral hepatitis among its 194 MS between 2012 and 2013. Findings were published in July 2013 in the *Global Policy Report on the Prevention and Control of Viral Hepatitis in WHO Member States* [23]. The quantitative data analyzed in this article are drawn from this survey. The data collection process for the survey is described in detail in Annex D of the report. In brief, a survey tool (Additional file 1) was developed for the purpose of collecting information about how national governments are addressing various aspects of viral hepatitis, including surveillance. After being piloted in 13 MS, it was distributed to all MS worldwide. In each MS, the national government focal point for viral hepatitis was asked to complete the survey. Data collection took place between July 2012 and February 2013. A total of 126 MS responded to the survey (65 %).

This article analyzes the data collected from the WHO European Region MS that participated in the survey. Responses were extracted for 18 questions pertaining to viral hepatitis surveillance; collection and use of other strategic information such as registries for death, liver cancer and HIV/hepatitis co-infection; viral hepatitis serosurveys; and screening of blood donors and pregnant women. We performed a descriptive analysis of all responses in accordance with the WHO European Region’s geographical divisions for its 53 MS (West, Centre and East), as well as a comparison between EU/EEA countries and non-EU/EEA countries.

EU/EEA membership was determined in accordance with member status at the end of 2013. Thirty MS were defined as EU/EEA countries, including Croatia, which was in the process of joining the EU at that time. Lichtenstein was excluded since it is not a WHO MS. Twenty-three WHO MS were non-EU/EEA countries.

Basic statistical analysis of the data was undertaken and differences between the EU/EEA and non-EU/EEA regions were analyzed using the Chi-squared test. Bivariate analyses were two-tailed with a significance level of 0.05. All analyses were carried out using IBM SPSS version 20.0 software (Armonk, NY, USA).

### Community hepatitis policy survey

In 2014, the World Hepatitis Alliance followed up the WHO policy survey with a global community hepatitis policy survey designed to give stakeholders outside of government the opportunity to share their perspectives. This survey, which was carried out by the University of Copenhagen, asked civil society organizations and community-based organizations, including patient groups, to assess the accuracy of their governments’ responses to the earlier WHO survey (Additional file 2). Respondents were also invited to comment further on hepatitis policy issues in their countries. Findings were published in July 2014 in the *Global Community Hepatitis Policy Report* [24].

This survey was written in English, and responses were sought from World Hepatitis Alliance members (patient groups) as well as other non-governmental organizations (NGOs), academic institutions and medical associations. The survey was distributed via e-mail to approximately 800 organizations worldwide identified by the World Hepatitis Alliance and the University of Copenhagen, including 253 organizations in the WHO European Region. Data collection took place between February 1, 2014 and June 15, 2014.

The first part of the survey requested information about the responding organization. The second part asked each respondent to review 25 items of information provided by the respondent’s country to the WHO survey, and to indicate whether he or she considered this information to be accurate. Respondents could also provide comments about each item. In the third part of the survey, respondents were invited to write statements about key national hepatitis policy issues of their choosing. Civil society stakeholders in countries where governments had not provided information to WHO were invited to complete a different survey that asked them to write statements about key national hepatitis policy issues of their choosing.

Responses from civil society organizations in the countries of the WHO European Region were collated for this study, and a descriptive analysis of the comments on surveillance-related questions was performed to identify key themes.

## Results

### WHO hepatitis policy survey

#### Respondents

**Fig. 1:**
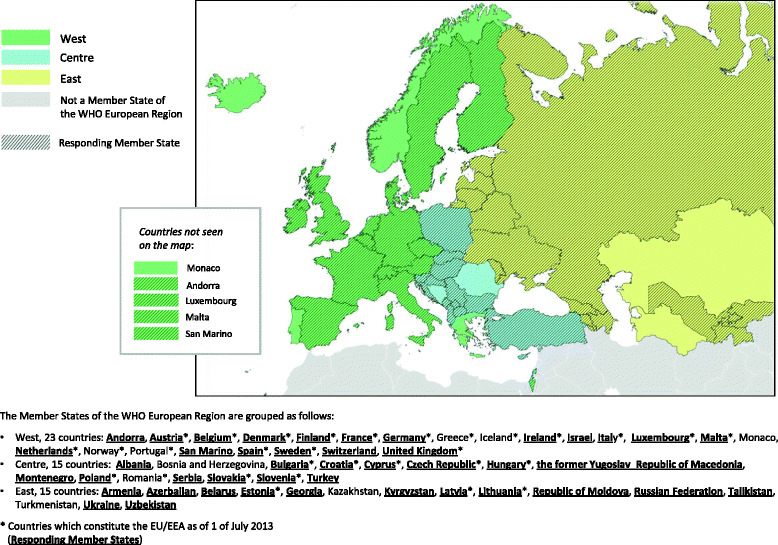
Geographical/epidemiological divisions of the WHO European Region

Forty-four (83 %) of the 53 MS in the WHO European Region replied to the survey and were included in the analysis. Across the three sub-regions, 18 of the 23 MS in the West responded (78 %), as did 13 of the 15 MS in the Centre (87 %) and 13 of the 15 MS in the East (87 %). The response rate was 83 % among both EU/EEA countries (25/30) and non-EU/EEA countries (19/23) (Fig. 1).

#### Descriptive findings

##### National coordination and routine surveillance

Thirteen responding MS (30 %) reported having a written national prevention and control strategy or plan for viral hepatitis: 5 (28 %) in the West, 3 (23 %) in the Centre and 5 (39 %) in the East. Twelve of those 13 MS (92 %) indicated that this strategy included a surveillance component (Table 1; Additional file 3). Ninety-eight percent of responding MS reported having national surveillance systems for acute hepatitis B, and 93 % for acute hepatitis C. Sixty-five percent of MS reported having a surveillance system for chronic hepatitis B, and 61 % for chronic hepatitis C. Ninety-six percent of MS reported having standard case definitions for viral hepatitis infections. Twenty-three percent responded that no viral hepatitis infections were reported to the national surveillance system as “undifferentiated” or “unclassified.” All responding MS reported having adequate laboratory capacity nationally to support hepatitis outbreak investigations and other surveillance activities for HBV and HBC. Finally, 89 % of responding MS reported that viral hepatitis disease reports were published at least once a year (Table 2; Additional file 4).

**Table 1:** Reporting by Member States on the existence of a written national viral hepatitis strategy or plan

	World Health Organization European sub-region			Total
	West	Centre	East	
	*N =* 18 (%)	*N =* 13 (%)	*N =* 13 (%)	*N =* 44 (%)
Existence of a written national strategy or plan that focuses exclusively or primarily on the prevention and control of viral hepatitis:				
* yes*	5 (27.8)	3 (23.1)	5 (38.5)	13 (29.6)
* no*	13 (78.2)	10 (76.9)	8 (61.5)	31 (70.5)
If there is a strategy or plan, does it include a surveillance component?				
	*N =* 5 (%)	*N =* 3 (%)	*N =* 5 (%)	*N =* 13 (%)
* yes*	5 (100)	3 (100)	4 (80.0)	12 (92.3)
* no*	0 (0)	0 (0)	1 (20.0)	1 (7.7)
If there is a strategy or plan, is it exclusive for viral hepatitis or does it also address other diseases?				
	*N =* 5 (%)	*N =* 3 (%)	*N =* 5 (%)	*N =* 13 (%)
* exclusive for viral hepatitis*	1 (20.0)	1 (33.3)	2 (40.0)	4 (30.8)
* only for HBV*	0 (0)	1 (33.3)	0 (0)	1 (7.7)
* only for HCV*	1 (20.0)	0 (0)	0 (0)	1 (7.7)
* integrated with other diseases*	3 (60.0)	1 (33.3)	3 (60.0)	7 (53.8)

**Table 2:** Reporting by Member States on routine surveillance of viral hepatitis B and C

	World Health Organization European sub-region			Total
	West	Centre	East	
	*N =* 18 (%)	*N =* 13 (%)	*N =* 13 (%)	*N =* 44 (%)
National surveillance system for acute HBV:				
* yes*	17 (94.4)	13 (100)	13 (100)	43 (97.3)
* no*	1 (5.6)	0 (0)	0 (0)	1 (2.3)
National surveillance system for acute HCV:				
* yes*	15 (83.3)	13 (100)	13 (100)	41 (93.2)
* no*	3 (16.7)	0 (0)	0 (0)	3 (6.8)
National surveillance system for chronic HBV:				
* yes*	11 (61.1)	7 (53.8)	10 (76.9)	28 (63.6)
* no*	7 (38.9)	6 (46.2)	3 (23.1)	16 (36.4)
National surveillance system for chronic HCV:				
* yes*	10 (55.6)	7 (53.8)	10 (76.9)	27 (61.4)
* no*	8 (44.4)	6 (46.2)	3 (23.1)	17 (38.6)
Standard case definitions for viral hepatitis infections:				
* yes*	17 (94.4)	13 (100)	12 (92.3)	42 (95.5)
* no*	1 (5.6)	0 (0)	1 (7.7)	2 (4.5)
% of hepatitis cases reported as “undifferentiated” or “unclassified”:				
* zero*	6 (33.3)	2 (15.4)	2 (15.4)	10 (22.7)
* less than 5 %*	2 (11.1)	4 (30.8)	3 (23.1)	9 (20.5)
* 5–15 %*	0 (0)	2 (15.4)	3 (23.1)	5 (11.4)
* more than 15 %*	1 (5.6)	2 (15.4)	1 (7.1)	4 (9.1)
* no response*	9 (50.0)	3 (23.1)	4 (30.8)	16 (36.4)
Adequate laboratory capacity nationally to support hepatitis outbreak investigations and other surveillance activities for HBV and HBC:				
* yes*	18 (100)	13 (100)	13 (100)	44 (100)
Hepatitis outbreaks required to be reported to the government and further investigated:				
* yes*	17 (94.4)	13 (100)	12 (92.3)	42 (95.5)
* no*	1 (5.6)	0 (0)	1 (7.7)	2 (4.5)
Hepatitis disease reports published regularly (at least once per year):				
* yes*	15 (83.3)	13 (100)	11 (84.6)	39 (88.6)
* no*	3 (16.7)	0 (0)	2 (15.4)	5 (11.4)

##### Other sources of strategic information and capacities for disease burden estimation

Ninety-six percent of MS responding to the survey reported having central registries for the reporting of deaths, including deaths related to viral hepatitis, and 80 % for the reporting of liver cancer cases. Sixty-six percent reported having national HIV/viral hepatitis co-infection registries. A total of 20 MS (46 %) reported undertaking viral hepatitis sero-surveys regularly. Target populations for sero-surveys most commonly included people who inject drugs (27 %), the general population (25 %), men who have sex with men (20 %) and pregnant women (20 %). Universal HBV screening for pregnant women was reported to take place in 77 % of responding MS. All countries with blood transfusion services reported screening all donated blood units for HCV infection, and all but Kyrgyzstan reported screening for HBV infection (Table 3; Additional file 5).

**Table 3:** Reporting by Member States on other sources of strategic information and capacities for disease burden estimation

	World Health Organization European sub-region			Total
	West	Centre	East	
	*N =* 18 (%)	*N =* 13 (%)	*N =* 13 (%)	*N =* 44 (%)
Deaths, including from hepatitis, reported to a central registry:				
* yes*	17 (94.4)	13 (100)	12 (92.3)	42 (95.5)
* no*	1 (5.6)	0 (0)	0 (0)	1 (2.3)
* no response*	0 (0)	0 (0)	1 (7.7)	1 (2.3)
Liver cancer cases registered nationally:				
* yes*	15 (83.3)	11 (84.6)	9 (69.2)	35 (79.5)
* no*	3 (16.7)	2 (15.4)	3 (23.1)	8 (18.2)
* do not know*	0 (0)	0 (0)	1 (7.7)	1 (2.3)
Cases of HIV/hepatitis co-infection registered nationally:				
* yes*	10 (55.6)	10 (76.9)	9 (69.2)	29 (65.9)
* no*	8 (44.4)	3 (23.1)	4 (30.8)	15 (34.1)
Viral hepatitis serosurveys conducted regularly:				
* yes*	8 (44.4)	5 (38.5)	7 (53.8)	20 (45.5)
* no*	10 (55.6)	6 (46.2)	6 (46.2)	22 (50.0)
* do not know*	0 (0)	2 (15.4)	0 (0)	2 (4.5)
Target populations for serosurveys (respondents were given the options of “children”, “general population”, “people who inject drugs”, “men who have sex with men”, and “other groups”, with space for identifying other groups):				
* children*	2 (11.1)	1 (7.7)	2 (15.4)	5 (11.4)
* general population*	4 (22.2)	5 (38.5)	2 (15.4)	11 (25.0)
* people who inject drugs*	4 (22.2)	3 (23.1)	5 (38.5)	12 (27.3)
* men who have sex with men*	2 (11.1)	4 (30.8)	3 (23.1)	9 (20.5)
* pregnant women*	2 (11.1)	2 (15.4)	5 (38.5)	9 (20.5)
* prisoners*	2 (11.1)	2 (15.4)	2 (15.4)	6 (13.6)
* health care workers*	0 (0)	2 (15.4)	4 (30.8)	6 (13.6)
* sex workers*	1 (5.6)	2 (15.4)	2 (15.4)	5 (11.4)
* people living with HIV*	0 (0)	1 (7.7)	2 (15.4)	3 (6.8)
* blood donors*	0 (0)	1 (7.7)	3 (23.1)	4 (9.1)
* military*	0 (0)	0 (0)	2 (15.4)	2 (4.5)
* Roma youth*	0 (0)	1 (7.7)	0 (0)	1 (2.3)
All pregnant women screened for HBV:				
* yes*	16 (88.9)	9 (69.2)	9 (69.2)	34 (77.3)
* no*	2 (11.1)	4 (30.8)	4 (30.8)	10 (22.7)
All donated blood (including family donations) screened for HBV:				
* Yes*	17 (94.4)	13 (100)	12 (100)	42 (95.5)
no	0 (0)	0 (0)	1 (7.7)	1 (2.3)
* N/A (no blood centres)*	1 (5.6)	0 (0)	0 (0)	1 (2.3)
All donated blood (including family donations) screened for HCV:				
* yes*	17 (94.4)	13 (100)	13 (100)	43 (97.7)
* N/A (no blood centres)*	1 (5.6)	0 (0)	0 (0)	1 (2.3)

##### Areas in which Member States might want assistance from WHO

Seventeen responding MS (39 %) indicated that they might want assistance from WHO in developing national viral hepatitis prevention and control plans, with the majority (*n =* 10; 77 %) of those MS located in the East. Ten countries (23 %) expressed interest in receiving WHO assistance with viral hepatitis surveillance, and 15 countries (34 %) did likewise for estimating the national burden of viral hepatitis. Again, much of the interest in receiving assistance came from the East, with eight MS (62 %) providing this response to both survey items (Table 4; Additional file 6).

**Table 4:** Reporting by Member States regarding areas in which they might want assistance from WHO

	World Health Organization European sub-region			Total
	West	Centre	East	
	*N =* 18 (%)	*N =* 13 (%)	*N =* 13 (%)	*N =* 44 (%)
Areas in which government might want WHO assistance:				
* national plan*	2 (11.1)	5 (38.5)	10 (76.9)	17 (38.6)
* surveillance*	1 (5.6)	1 (7.7)	8 (61.5)	10 (22.7)
* burden estimation*	1 (5.6)	6 (46.2)	8 (61.5)	15 (34.1)

#### Differences in survey findings between EU/EEA and non-EU/EEA member states

With regard to routine surveillance, slightly more countries in the EU/EEA than in non-EU/EEA MS reported conducting surveillance for chronic hepatitis, and all EU/EEA countries had standard case definitions, but these differences were not statistically significant. There were also non-significant differences regarding liver cancer registries and central death registries, with slightly more EU/EEA MS reporting that both were in place in their countries. Statistically significant differences occurred for only two survey items. More non-EU/EEA countries expressed interest in two forms of assistance from WHO: assistance developing national plans for viral hepatitis prevention and control (58 % versus 24 %, *p =* 0.024); and assistance with surveillance (42 % versus 8 %, *p =* 0.010) (Table 5).

**Table 5:** Member States of the WHO European region reporting on surveillance-related activities by EU/EEA membership

	EU/EEA		non-EU/EEA		
	*N =* 25	%	*N =* 19	%	p-value
National coordination					
Existence of a written national strategy or plan that focuses exclusively or primarily on the prevention and control of viral hepatitis and includes a surveillance component	6	24.0	6	31.6	0.412
Routine surveillance of viral hepatitis B and C					
National surveillance system for acute HBV	25	100	18	94.7	0.432
National surveillance system for acute HCV	23	92.0	18	94.7	0.604
National surveillance system for chronic HBV	18	72.0	10	52.6	0.157
National surveillance system for chronic HCV	17	68.0	10	52.6	0.234
Standard case definitions for viral hepatitis infections	25	100	17	89.5	0.181
Regular hepatitis disease reports published	22	88.0	17	89.5	0.632
Hepatitis outbreaks required to be reported to the government and further investigated	23	92.0	19	100	0.317
Adequate laboratory capacity nationally to support hepatitis outbreak investigations and other surveillance activities for HBV and HBC	25	100	19	100	-
Other sources of strategic information and capacities for disease burden estimation					
Deaths, including from hepatitis, reported to a central registry	25	100	17	89.5	0.181
Liver cancer cases registered nationally	22	88.0	13	68.4	0.122
Cases of HIV/hepatitis co-infection registered nationally	16	64.0	13	68.4	0.508
Viral hepatitis serosurveys conducted regularly	10	40.0	10	52.6	0.299
If yes, please specify the target populations:					
- children	3	12.0	2	10.5	0.632
- general population	8	32.0	3	15.8	0.191
- people who inject drugs	6	24.0	6	31.6	0.412
- men who have sex with men	3	12.0	6	31.6	0.112
- other groups	7	28.0	10	52.6	0.089
All pregnant women screened for HBV	21	84.0	13	68.3	0.195
All donated blood (including family donations) screened for HBV	25	100	17^a^	94.4	0.419
All donated blood (including family donations) screened for HCV	25	100	18^a^	100	0.432
Areas in which Member States might want assistance from WHO					
National plan	6	24.0	11	57.9	0.024
Surveillance	2	8.0	8	42.1	0.010
Burden estimation	6	24.0	9	47.4	0.097

^a^Andorra is not included in this calculation because Andorra does not have blood donation centres

### Global community hepatitis policy survey

Forty organizations from 27 countries in the WHO European Region responded to the World Hepatitis Alliance’s global community policy survey. Fifteen organizations (38 %) identified themselves as hepatitis patient groups and another seven (18 %) identified themselves as NGO direct service providers. Other types of organizations included other types of NGOs as well as medical societies and private foundations. Twenty-three of these countries (12 from the countries outside of the EU/EEA) had responded to the 2013 WHO global policy survey, and thus, the 35 respondents based in those countries were able to comment on the accuracy of their governments’ responses.

In their comments, a number of respondents disputed or questioned the information that their governments had reported to WHO about surveillance policies and practices. Areas of disagreement related to the existence of routine viral hepatitis surveillance, the registration of liver cancer cases and cases of HIV/hepatitis co-infection, and the publication of hepatitis disease reports.

*There is definitely no national registry for hepatocellular cancer. The information provided [for the WHO survey] is a misunderstanding, because it looks like the government representative who completed the survey confused the voluntary reporting system that exists in Poland with a register which contains all crucial data about particular patients. Of course data on hepatocellular carcinoma and hepatitis patients are collected by the National Health Fund but they are not analyzed and not provided upon the request of medical societies or even pharmaco-economic agencies.* – Polish Association for the Study of the Liver (Poland)

*I have never seen a hepatitis disease report from government. Very curious to [know what the survey submitted to WHO] means regarding a “hepatitis disease report.”* – Associazione EpaC (Italy)

Additionally, some respondents indicated that, although their governments had reported having surveillance systems in place, the systems did not function adequately with regard to distinguishing between acute and chronic viral hepatitis or capturing other important information.

*There is an inefficient [surveillance] system, which has been improved recently by shifting the reporting from the physicians to the virology laboratories. However, there is no information available on the clinical scenario, and no distinction is possible between acute and chronic hepatitis. Also, this is not routine surveillance in a strict sense but just opportunistic surveillance, finding cases by chance if the treating physician decides to order a test.* – Österreichische Gesellschaft für Gastroenterologie und Hepatologie (Austria)

*This is a misunderstanding. Government recognizes as a “surveillance system” the voluntary reporting of hepatitis cases by physicians. So this is a passive system. There is no active surveillance program based on the screening of high-risk populations. … The data collected by the National Institute of Health – recognized as a surveillance system – provides just a reporting rate and not a prevalence rate.* – Polish Association for the Study of the Liver (Poland)

*In a case when someone died from cirrhosis, the autopsy says “death from cirrhosis” but it is never specified whether it was caused by hepatitis and what kind.* – National Association for Fighting Hepatitis – Hepasist (Bulgaria)

Some respondents expressed concern about surveillance systems not gathering information on treatment uptake or treatment coverage.

*In England noone knows how many people are treated each year for HCV or HBV or what the outcomes are, an extraordinary situation given the cost. – The Hepatitis C Trust (United Kingdom)*

*The governmental structures receive partial information on acute hepatitis, and the information is spread by word-of-mouth. There are no follow-up data on what happens to those diagnosed with acute hepatitis and whether they receive treatment. – National Association for Fighting Hepatitis* – Hepasist (Bulgaria)

## Discussion

This sub-analysis of surveillance-related data from the WHO *Global Policy Report on the Prevention and Control of Viral Hepatitis in WHO Member States* demonstrates that key surveillance components are in place in most MS, suggesting that there is considerable potential to acquire better data for decision-making. However, substantial systemic shortcomings in hepatitis B and C surveillance were reported throughout the WHO European Region. Survey findings for EU/EEA and non-EU/EEA MS did not differ significantly for the most part, suggesting that attention should be given to strengthening surveillance and strategic information systems across the entire region.

While more than 90 % of MS reported conducting surveillance for acute HBV and HCV infections, the surveillance of chronic HBV and HCV infections was less common. One reason for this may be that viral hepatitis surveillance systems historically have focused on collecting data on acute infections, primarily for the purpose of identifying outbreaks. When WHO last published recommendations on viral hepatitis surveillance in 2003, the recommendations addressed surveillance of only acute infection [25]. Another influential source of technical guidance for the EU/EEA countries, ECDC, has only distinguished chronic infection with a revision of the EU case definitions for hepatitis B and C surveillance in conjunction with the recent implementation of its enhanced surveillance program. Data collected by the ECDC from the 31 countries it serves indicated that in 2013, only 17 countries were able to provide data on chronic HBV infection [26], and 12 on chronic HCV infection [5]. The ECDC experience, coupled with our own findings, suggests that surveillance systems may not be evolving rapidly enough to keep pace with recent developments in viral hepatitis prevention and treatment. A greater orientation toward chronic disease surveillance would contribute to efforts to understand the hepatitis disease burden, assess the impact of prevention and treatment efforts, and maximize the impact of resources [27].

The classification of viral hepatitis infection as acute versus chronic is a widely recognized challenge, especially for hepatitis C [28]. Data from our study indicate that only 10 MS (23 %) have no hepatitis cases reported as “undifferentiated” or “unclassified.” Some community survey respondents’ comments provided further evidence that distinguishing between acute and chronic hepatitis might not be possible in their countries. While most countries have case definitions, these vary across countries, making it difficult to compare data between countries. Progress has been made in the EU/EEA with the 2012 adoption of the revised EU case definitions [29], but further work is needed in this area, including the development of more accurate serological tests to better distinguish between acute and chronic HCV infection, and, to a lesser extent, for HBV infection as well.

All responding countries stated that they have adequate laboratory capacity nationally for outbreak investigations and other surveillance activities related to HBV and HCV, an indication that there might be considerable potential to improve and enhance viral hepatitis surveillance, including electronic-based reporting from laboratories. The survey information, however, should be regarded cautiously, as laboratory capacity at the central level might differ substantially from laboratory capacity at the regional and local levels.

The viral hepatitis disease burden can be assessed more accurately when data from case reporting are considered in conjunction with data from other sources such as death registries and disease registries. Almost all responding MS in our study reported having central death registries. Although these can potentially be used to assess the proportions of deaths attributable to HBV and HCV, the contribution of viral hepatitis to chronic liver disease-related mortality may be underestimated if death records are the only source of information [30, 31]. Since most deaths from HBV and HCV are due to complications of liver disease rather than being directly attributable to the viral infection, viral hepatitis may not be specified on the death certificate. Nonetheless, the usefulness of death registries in assessing the HBV and HCV disease burden and evaluating the population-level impact of treatment could be improved by linking such registries to viral hepatitis diagnosis databases [32]. Disease registries are another potentially valuable source of hepatitis information. In Europe, up to 15 % of primary liver cancer cases are caused by HBV, and up to 70 % by HCV [33]. The majority of European countries responding to the WHO survey reported the existence of national liver cancer registries, but the use of these data might be problematic, as noted by a community survey respondent from Poland. The feasibility of using liver cancer incidence indicators associated with cancer registries for evaluating the burden of chronic viral hepatitis infection has been demonstrated [34], but the accurate contribution of HBV and HCV infection to worldwide cancer trends is not well defined [35].

Two-thirds (66 %) of responding MS reported having national registries for HIV/viral hepatitis co-infection. Such databases can be used as additional data sources for chronic hepatitis surveillance and for identifying high-prevalence groups that should be targeted for screening [36]. In most jurisdictions globally, comprehensive HIV/AIDS surveillance has been in place for many years. The ECDC and the WHO Regional Office for Europe have effectively collaborated to jointly coordinate HIV/AIDS surveillance since 2008 for all 53 countries in the WHO European Region, although data on HIV/viral hepatitis co-infection are not currently being reported at the regional level [37]. The example of the joint ECDC/WHO surveillance for HIV/AIDS may serve as a useful model for strengthening viral hepatitis surveillance at the regional level in the future.

Data from sero-surveys can make a unique contribution to assessments of the burden of HBV and HCV disease because sero-surveys identify the many asymptomatic undiagnosed infections. Systematically repeated sero-surveys can be considered a form of surveillance, while single surveys provide a baseline measure of disease prevalence. In addition, the results can be compared and integrated with simultaneously collected epidemiologic information or case reporting data [38]. Only 20 of 44 (45 %) responding study countries reported conducting viral hepatitis sero-surveys regularly, and three other countries (Croatia, Czech Republic and Latvia) reported that they had conducted sero-surveys at least occasionally. It is likely that the complexity and expense of conducting sero-surveys are both factors limiting their role in HBV and HCV surveillance in the WHO European Region.

Many national surveillance programs may be able to make good use of HBV and HCV seroprevalence data that are already being recorded for two populations: blood donors and pregnant women. Both populations are subject to routine testing in accordance with health care standards in many countries. In our study, almost all responding MS reported having routine blood donor screening for both HBV and HCV. In most cases, data on the prevalence of HBV and HCV among blood donors is readily available, thus providing a basis for estimating seroprevalence within the general population. However, this is not a good proxy in most countries due to pre-screening selection of donors. HBV and HCV seroprevalence data for pregnant women may be more informative, but these data are not always as readily available as data from blood transfusion services because of less strictly regulated reporting from maternity services to public health officials. Furthermore, far fewer countries screen pregnant women for HCV than HBV [6]. In our study, 77 % of responding MS reported having routine HBV screening for pregnant women for HBV.

The lack of statistically significant differences between EU/EEA countries and non-EU/EEA countries in regard to the existence of chronic hepatitis surveillance and national death and liver cancer registries was somewhat surprising considering that EU/EEA countries generally have higher income levels and greater health spending. Twenty-two responding EU/EEA MS (88 %) were high-income countries, while 15 non-EU/EEA respondents (79 %) were low/middle income countries (*p <* 0.001), as determined by World Bank 2012 country income classifications. Also, EU/EEA countries might be expected to benefit from the ECDC’s ongoing work to harmonize and improve viral hepatitis surveillance in the region that it serves. The finding that non-EU/EEA countries were interested in potential assistance from WHO on national planning and surveillance-related activities could be partly explained by the fact that countries in Eastern Europe and Central Asia traditionally have received technical assistance from WHO, and thus may be more likely to request it in the future. At the same time, the finding indirectly suggests that their surveillance systems may need greater attention than those in EU/EEA countries.

Responses from the global community hepatitis policy survey raise the following issues. First, some disagreements with government information regarding national surveillance policies and practices may indicate areas in which governments misreported facts for the WHO global policy survey. It is also possible that civil society respondents may have questioned the accuracy of some government information that was actually correct but was not sufficiently communicated to all relevant stakeholders. For example, civil society stakeholders in two countries made statements questioning their governments’ assertions that hepatitis disease reports are published. One explanation may be that government agencies are compiling and disseminating reports internally but not making people outside of those agencies aware of their existence. Beyond civil society respondents’ concerns about the accuracy of what their governments reported, some of their statements appear to offer informed opinions about surveillance issues. While public health surveillance by its nature is largely, or entirely, carried out by governments, findings from this study suggest that civil society stakeholders in some settings may be able to make important contributions to strengthening viral hepatitis surveillance. Governments should consider this point as they take action on the World Health Assembly’s 2014 viral hepatitis resolution, which, among other points, urges MS “to promote the involvement of civil society in all aspects of preventing, diagnosing and treating viral hepatitis” [17].

In sum, virtually all responding countries in the WHO European Region reported having viral hepatitis surveillance systems in place, and there seem to be similar challenges and opportunities for improvement across the Region. However, it should be emphasized that the WHO survey collected information about the existence of policies and practices from national focal points but did not assess the extent or quality of implementation. Thus, it would be premature to draw conclusions about the overall effectiveness or ineffectiveness of national viral hepatitis surveillance systems based on the findings of this study. Furthermore, before such systems can be rigorously assessed, clarity is needed regarding the aims, outcomes and capacities of surveillance. It may be that many countries are achieving their stated viral hepatitis surveillance aims even if they do not have all of the components of what might be considered an ideal surveillance system. It would be beneficial for the WHO European Region as a whole to have a shared understanding of the specific ways in which surveillance is intended to support effective responses to HBV and HCV. This approach might entail recognizing, for example, the importance of surveillance data for developing estimates of adverse outcomes of HBV and HCV, such as those presented in the Global Burden of Disease study [39], even while the surveillance data are understood to provide an incomplete picture. It also might entail adopting common definitions in order to avoid confusion about issues such as those brought to light by the Polish civil society respondent who asserted that, what the government considers to be a surveillance system, is merely a record of physicians’ voluntary reporting of hepatitis cases. The prospect of large numbers of people with chronic HCV gaining access to new treatments with high cure rates in the coming years further underscores the need for clarification about what national surveillance systems can contribute to tracking the changing epidemiology of this disease.

### Limitations

Our study suffers from a number of limitations in addition to those already identified. First, although the European Region had the second-highest response rate to the WHO global policy survey among the six WHO regions, the absence of information from nine European MS (13 %) limits the generalizability of study findings. Other limitations relate to language and terminology. Survey respondents were restricted to using five languages (English, French, Spanish, Portuguese and Russian), and this may have resulted in some respondents not fully comprehending all of the survey questions. Furthermore, some questions in the survey might have been understood differently by respondents in different settings. Finally, the data in the WHO *Global Policy Report* were submitted by the identified focal points from MS, and researchers did not verify the accuracy of the data prior to publication of the report.

To some extent, the World Hepatitis Alliance’s civil society survey provides useful complementary information, although that survey was not designed to verify governments’ responses to individual survey items. Also, many respondents may not be fully qualified to have a complete overview of the national surveillance system and to assess whether responses to the WHO survey were indeed correct. The civil society survey has additional limitations as a robust source of data. Survey responses were received from only about half of all European countries, and some countries were represented by a single civil society respondent while others were represented by multiple civil society respondents. Furthermore, organizations that disagreed with the data provided by governments may have been more likely to respond to the survey than organizations that agreed. These factors limit the extent to which generalizations can be made on the basis of specific civil society survey findings.

## Conclusions

To conclude, our study indicates that there is high overall capacity for robust HBV and HCV surveillance throughout the WHO European Region, but that existing surveillance systems have substantial weaknesses. Many governments have surveillance systems in place, along with laboratory capacity, and many governments are producing surveillance reports on at least an occasional basis. However, findings from the WHO policy survey and the follow-up civil society survey point to shortcomings, such as a lack of surveillance for chronic HBV and HCV disease, a lack of serosurveillance and an absence of mechanisms for tracking treatment outcomes in many MS. International organizations such as WHO and ECDC have the potential to play an important role in supporting country-level efforts to improve viral hepatitis surveillance and developing a comprehensive framework for the collection, analysis and use of strategic information. There is an urgent need for European countries to strengthen their surveillance programs to be able to monitor national progress toward achieving ambitious global targets relating to the elimination of viral hepatitis and to maximize the population-level impact of advances in HBV and HCV prevention and treatment.

## Abbreviations

CDC, centres for disease control; ECDC, European centre for disease prevention and control; EEA, European economic area; EFTA, European free trade association; EU, European union; HBsAg, hepatitis B surface antigen; HBV, hepatitis B virus; HCV, hepatitis C virus; MS, member states; NGOs, non-governmental organizations; WHO, World Health Organization

## Additional files

Additional file 1: Annex E: Responding to viral hepatitis: the World Health Organization/World Hepatitis Alliance 2012 survey of national governments. (PDF 85 kb)

Additional file 2: Annex B: World Hepatitis Alliance 2014 Survey of Civil Society Stakeholders. (PDF 114 kb)

Additional file 3: Reporting by Member States on the existence of a written national viral hepatitis strategy or plan. (DOCX 30 kb)

Additional file 4: Reporting by Member States on routine surveillance of viral hepatitis B and C. (DOCX 36 kb)

Additional file 5: Reporting by Member States on other sources of strategic information and capacities for disease burden estimation. (DOCX 39 kb)

Additional file 6: Reporting by Member States regarding areas in which they might want assistance from WHO. (DOCX 25 kb)
