# Supplementary material for: Strengthening hepatitis B and C surveillance in Europe: results from the two global hepatitis policy surveys (2013 and 2014)
Source: Hepatol Med Policy. 2016 Jun 30;1:3. doi: 10.1186/s41124-016-0009-5 (PMC5918699; doi:10.1186/s41124-016-0009-5)
Supplement: Supplementary file 1 — Annex E: Responding to viral hepatitis: the World Health Organization/World Hepatitis Alliance 2012 survey of national governments. (PDF 85 kb) [file 41124_2016_9_MOESM1_ESM.pdf]

ANNEX E: Responding to viral hepatitis: the World Health Organization/World Hepatitis Alliance 2012 survey of national governments

Your cooperation is requested in gathering data that will enable the World Health Organization and the World Hepatitis Alliance to assess the response to viral hepatitis at the global, regional and national levels.

This information is vital for measuring progress, identifying gaps and guiding future efforts. Please fully answer all questions in English. You may provide supporting information in any language. If you do not know the answer to a question, please make every effort to find the information. Please submit the completed survey, along with all supporting information, to obaran@who.int with a cc to hepatitis@cphiv.dk.

Contact information

First name ______________________

Last name ______________________

Position ______________________

Organization ______________________

Street address ______________________

City ______________________

Postal code / zip code ______________________

Country ______________________

Phone number ______________________

E-mail address ______________________

Web site ______________________

Where appropriate, please give the names and contact details of colleagues who provided supporting information.

1. In your country, is there a ***written*** national strategy or plan that focuses exclusively or primarily on the prevention and control of viral hepatitis?[a]

( ) yes ( ) no ( ) do not know

[a] Please e-mail us the relevant supporting information, including if you have a strategy or plan for other diseases which includes viral hepatitis.

If yes –

Is the strategy or plan exclusive for viral hepatitis or does it also address other diseases (e.g. HIV, sexually transmitted infections)?

a. ( ) exclusive for viral hepatitis

b. ( ) only for hepatitis B

c. ( ) only for hepatitis C

d. ( ) integrated with other diseases

e. ( ) do not know

Please indicate whether the following are components of the strategy or plan:

1. Raising awareness	( ) yes	( ) no	( ) do not know
2. Surveillance	( ) yes	( ) no	( ) do not know
3. Vaccination	( ) yes	( ) no	( ) do not know
4. Prevention in general	( ) yes	( ) no	( ) do not know
5. Prevention of transmission via injecting drug use	( ) yes	( ) no	( ) do not know
6. Prevention of transmission in health-care settings	( ) yes	( ) no	( ) do not know
7. Treatment and care	( ) yes	( ) no	( ) do not know
8. Coinfection with HIV	( ) yes	( ) no	( ) do not know

2. Is there a designated governmental unit/department responsible only for coordinating and/or carrying out viral hepatitis-related activities?

( ) yes ( ) no ( ) do not know

a. If yes, how many staff members does it have? __________

b. Name of unit/department ______________________

3. How many people work full-time (or how many full-time equivalent staff) on hepatitis-related activities in all government agencies/bodies?

__________ people ( ) do not know

4. Did your government hold events for World Hepatitis Day 2012?[a]

( ) yes ( ) no ( ) do not know

[a] Please e-mail us the relevant supporting information.

5. Has your government funded any public viral hepatitis awareness campaign since January 2011, other than World Hepatitis Day?[a]

( ) yes ( ) no ( ) do not know

[a] Please e-mail us the relevant supporting information (including the names of the campaigns).

If yes, what were the primary topics or messages of the campaigns?

a. ( ) General information about hepatitis and its transmission

b. ( ) Vaccination for hepatitis A and hepatitis B

c. ( ) Importance of knowing one's hepatitis B and hepatitis C status

d. ( ) Safe water and good sanitation

e. ( ) Safer sex practices

f. ( ) Harm reduction for people who inject drugs

g. ( ) Safe workplace practices

h. ( ) Other – please specify:

a. __________

b. __________

c. __________

6. Does your government have a viral hepatitis prevention and control programme that includes activities targeting specific populations?

( ) yes ( ) no ( ) do not know

If yes, please indicate which populations:

a. ( ) Health workers (including health-care waste handlers)

b. ( ) People who inject drugs

c. ( ) Migrants

d. ( ) Prisoners

e. ( ) The homeless

f. ( ) People living with HIV

g. ( ) Low-income populations

h. ( ) The uninsured

i. ( ) Indigenous people

j. ( ) Other - (please specify): __________

7. Does your government collaborate with any civil society group within your country (such as patient groups or national or local nongovernmental organizations) to develop and implement its viral hepatitis prevention and control programme? **If yes,** please name major partners.

( ) yes ( ) no major partners

a. __________

b. __________

c. __________

8. Is there routine surveillance for viral hepatitis?

( ) yes ( ) no ( ) do not know

If yes, please answer the following questions regarding viral hepatitis surveillance. If no, please proceed to Question 11.

9. Is there a national surveillance system for the following types of ***acute*** viral hepatitis infection? Please check all that apply.

( ) yes ( ) no ( ) do not know

a. Hepatitis A ( ) yes ( ) no ( ) do not know

b. Hepatitis B ( ) yes ( ) no ( ) do not know

c. Hepatitis C ( ) yes ( ) no ( ) do not know

d. Hepatitis D ( ) yes ( ) no ( ) do not know

e. Hepatitis E ( ) yes ( ) no ( ) do not know

10. Is there a national surveillance system for ***chronic*** hepatitis infection? Please check all that apply.

( ) yes ( ) no ( ) do not know

a. Hepatitis B ( ) yes ( ) no ( ) do not know

b. Hepatitis C ( ) yes ( ) no ( ) do not know

c. Hepatitis D ( ) yes ( ) no ( ) do not know

If yes, please e-mail us the definitions and/or source used.

11. Are there standard case definitions for hepatitis infections?

( ) yes ( ) no ( ) do not know

12. Are deaths, including from hepatitis, reported to a central registry?

( ) yes ( ) no ( ) do not know

13. What percentage of hepatitis cases are reported as "undifferentiated" or "unclassified" hepatitis?

( ) yes ( ) no ( ) do not know

14. Are liver cancer cases registered nationally?

( ) yes ( ) no ( ) do not know

15. Are cases with HIV/hepatitis coinfection registered nationally?

( ) yes ( ) no ( ) do not know

16. How often are hepatitis disease reports published?

a. ( ) weekly b. ( ) monthly c. ( ) annually

d. ( ) no reports published e. ( ) other: __________

17. Are hepatitis outbreaks required to be reported to the government?

( ) yes ( ) no ( ) do not know

a. **If yes,** are they further investigated?

( ) yes ( ) no ( ) do not know

18. Is there a national public health research agenda for viral hepatitis?[a]

( ) yes ( ) no ( ) do not know

[a] Please e-mail us the relevant documentation, including information about the budget and networks involved.

19. Are viral hepatitis serosurveys conducted regularly?

( ) yes ( ) no ( ) do not know

If yes –

a. How often? __________

b. When was the last one carried out? __________

c. Please specify the target populations:

1. ( ) Children (please specify age group): __________
2. ( ) General population
3. ( ) People who inject drugs
4. ( ) Men who have sex with men
5. ( ) Other groups: __________

20. Is there adequate laboratory capacity nationally to support investigation of viral hepatitis outbreaks and other surveillance activities?

( ) yes ( ) no ( ) do not know

a. Hepatitis A ( ) yes ( ) no ( ) do not know

b. Hepatitis B ( ) yes ( ) no ( ) do not know

c. Hepatitis C ( ) yes ( ) no ( ) do not know

d. Hepatitis E ( ) yes ( ) no ( ) do not know

21. Is there a national hepatitis A vaccination policy?

( ) yes ( ) no ( ) do not know

If yes, does the policy address vaccination for the following groups? Please check all that apply.

a. ( ) Travellers to highly endemic countries

b. ( ) Military personnel

c. ( ) Children, as part of the national routine vaccination programme

d. ( ) Ecological and sanitary workers

e. ( ) Other – please specify: __________

22. Has your government established the goal of eliminating hepatitis B?

( ) yes ( ) no ( ) do not know

a. **If yes,** in what timeframe (e.g. an end date)?

____

23. Nationally, what percentage of newborn infants in a given recent year received the first dose of hepatitis B vaccine within 24 hours of birth?

____

24. Nationally, what percentage of one-year-olds (ages 12–23 months) in a given recent year received three doses of hepatitis B vaccine?

____

25. Nationally, what percentage of one-year-olds (ages 12–23 months) in a given recent year received three doses of hepatitis B vaccine?

Is there a national policy that specifically targets mother-to-child transmission of hepatitis B? Please check the following if the policy calls for any of these activities:

a. ( ) All pregnant women are screened for hepatitis B

b. ( ) All pregnant women found to have hepatitis B are counselled

c. ( ) Health-care providers follow up with all pregnant women found to have hepatitis B during pregnancy for the purpose of encouraging them to give birth at health-care facilities

d. ( ) Upon delivery, all infants born to women with hepatitis B receive hepatitis B immunoglobulin

e. ( ) All infants receive the first dose of hepatitis B vaccine within 24 hours of birth

f. ( ) All infants receive the second and third doses of hepatitis B vaccine within 12 months of birth

26. Is there a specific national strategy and/or policy/guidelines for preventing hepatitis B and hepatitis C infection in health-care settings?

( ) yes ( ) no ( ) do not know

a. If yes, are health workers vaccinated against hepatitis B prior to starting work that might put them at risk of exposure to blood?

( ) yes ( ) no ( ) do not know

27. Is there a national policy on injection safety in health-care settings?

( ) yes ( ) no ( ) do not know

a. If yes, what type of syringes does the policy recommend for therapeutic injections?

1. ( ) single-use syringes

2. ( ) auto-disable syringes

3. ( ) do not know

28. Are single-use or auto-disable syringes, needles and cannulas always available in all health-care facilities?

( ) yes ( ) no ( ) do not know

29. What are your government's official estimates of the number and percentage of unnecessary injections administered annually in health-care settings? (e.g. injections that are given when an equivalent oral medication is available)

a. Number ____________ ( ) do not know

b. Percentage ____________ ( ) do not know
(as a proportion of total injections administered annually in health-care settings)

30. Is there a national infection control policy for blood banks?[a]

( ) yes ( ) no ( ) do not know

[a] Please e-mail us the relevant supporting information, including the percentage of blood donations screened for hepatitis B virus, hepatitis C virus and HIV.

31. Are all donated blood units (including family donations) and blood products nationwide screened for hepatitis B?

( ) yes ( ) no ( ) do not know

32. Are all donated blood units (including family donations) and blood products nationwide screened for hepatitis C?

( ) yes ( ) no ( ) do not know

33. Is there a national policy relating to the prevention of viral hepatitis among people who inject drugs?[a]

( ) yes ( ) no ( ) do not know

[a] Please e-mail us the relevant supporting information.

34. Does your government have guidelines that address how hepatitis A and hepatitis E can be prevented through food and water safety?

( ) yes ( ) no ( ) do not know

35. How do health professionals in your country obtain the skills and competencies required to effectively care for people with viral hepatitis?

a. ( ) schools for health professionals (pre-service education)

b. ( ) on-the-job training

c. ( ) postgraduate training

d. ( ) other: ____________

e. ( ) do not know

36. Does your government have a national policy relating to screening and referral to care for the following?[a]

( ) yes ( ) no ( ) do not know

a. Hepatitis B ( ) yes ( ) no ( ) do not know

b. Hepatitis C ( ) yes ( ) no ( ) do not know

[a] Please e-mail us the relevant supporting information.

37. Please answer the following questions about hepatitis B and hepatitis C testing in your country. Please check the appropriate boxes.

	a. Hepatitis B	b. Hepatitis C
1. When testing, do people register by name?	( ) yes<br>( ) no	( ) yes<br>( ) no
2. If people register by name, are their names kept confidential within the system, or is there open access to the names?	( ) confidential<br>( ) open access	( ) confidential<br>( ) open access
3. Is the test free of charge for all individuals?	( ) yes<br>( ) no	( ) yes<br>( ) no
4. Is the test free of charge for members of any specific group?	( ) yes (please indicate):<br>( ) no	( ) yes (please indicate):<br>( ) no
5. Is the test compulsory for members of any specific group?	( ) yes (please indicate):<br>( ) no	( ) yes (please indicate):<br>( ) no

38. Are there national clinical guidelines for the management of viral hepatitis?[a]

( ) yes ( ) no ( ) do not know

[a] Please e-mail us the relevant supporting information.

a. **If yes,** do they include recommendations for cases with HIV coinfection?

( ) yes ( ) no ( ) do not know

b. **If no,** are there national clinical guidelines for the management of HIV,which include recommendations for coinfection with viral hepatitis?

( ) yes ( ) no ( ) do not know

39. Is publicly funded treatment available for hepatitis B?

( ) yes ( ) no ( ) do not know

a. If yes, who is eligible for publicly funded treatment for hepatitis B? Please specify:

____

40. Is publicly funded treatment available for hepatitis C?

( ) yes ( ) no ( ) do not know

a. If yes, who is eligible for publicly funded treatment for hepatitis C? Please specify:

____

41. How much does the government spend on publicly funded treatment for hepatitis B and hepatitis C? (Please indicate currency. Please also indicate whether the amount is for one or both of the infections.)

____

____

42. Which of the following drugs for treating hepatitis B and hepatitis C are on the national essential medicines list or subsidized by the government? (Please check all that apply.)

a. Available drugs for treating hepatitis B:

1. ( ) Interferon alpha
2. ( ) Pegylated interferon
3. ( ) Lamivudine (Epivir-HBV, Zeffix or Heptodin)
4. ( ) Adefovir dipivoxil (Hepsera)
5. ( ) Entecavir (Baraclude)
6. ( ) Telbivudine (Tyzeka, Sebivo)
7. ( ) Tenofovir (Viread)
8. ( ) Other: ______________________________

b. Available drugs for treating hepatitis C:

1. ( ) Interferon alpha
2. ( ) Pegylated interferon
3. ( ) Ribavirin
4. ( ) Boceprevir (Victrelis)
5. ( ) Telaprevir (Incivo, Incivek)
6. ( ) Other: ______________________________

43. Please indicate in which areas, if any, your government might want assistance from the World Health Organization for the prevention and control of viral hepatitis:

a. ( ) Developing the national plan for viral hepatitis prevention and control

b. ( ) Viral hepatitis surveillance

c. ( ) Increasing coverage of the birth dose of the hepatitis B vaccine

d. ( ) Estimating the national burden of viral hepatitis

e. ( ) Developing tools to assess the effectiveness of interventions

f. ( ) Increasing access to treatment

g. ( ) Increasing access to diagnostics

h. ( ) Improving laboratory quality

i. ( ) Awareness-raising

j. ( ) Developing education/training programmes for health professionals

k. ( ) Assessing the economic impact of viral hepatitis

l. ( ) Integrating viral hepatitis programmes into other health services

m. ( ) Other – please specify:
