# Supplementary material for: Strengthening hepatitis B and C surveillance in Europe: results from the two global hepatitis policy surveys (2013 and 2014)
Source: Hepatol Med Policy. 2016 Jun 30;1:3. doi: 10.1186/s41124-016-0009-5 (PMC5918699; doi:10.1186/s41124-016-0009-5)
Supplement: Supplementary file 2 — Annex B: World Hepatitis Alliance 2014 Survey of Civil Society Stakeholders. (PDF 114 kb) [file 41124_2016_9_MOESM2_ESM.pdf]

Annex B: World Hepatitis Alliance 2014 Survey of Civil Society Stakeholders

This annex presents the two surveys that form the basis of this report: the first was for organisations in countries where governments contributed information to the 2013 WHO hepatitis policy survey and the second was for organisations in countries where governments did not contribute information.

World Hepatitis Alliance 2014 Survey of Civil Society Stakeholders

A response to the Global Policy Report on the Prevention and Control of Viral Hepatitis in WHO Member States (World Health Organization, 2013)

Your organisation's cooperation is requested in gathering data for the World Hepatitis Alliance's 2014 global hepatitis policy report. This report is seen as a civil society response to information provided by governments for the 2013 global hepatitis policy report published by the World Health Organization (WHO) (http://www.who.int/csr/disease/hepatitis/global_report/en/).

The following survey has three parts:

- In **Part A** your organisation is asked to provide basic information about itself.
- In **Part B** your organisation is asked to review the actual published text that describes what your government stated for the 2013 report, and to comment on whether the information is correct.
- In **Part C** your organisation is asked to discuss the policy response to hepatitis in your country in greater depth, focusing on issues that your organisation wishes to prioritise. Your organisation also is invited to put forth an "agenda for change" outlining proposed roles and responsibilities for key stakeholders.

Part A. Organisational information

Organisation name ______

Street address ______

City ______ Postal code/zip code ______

Country ______

Website ______

First name of person completing survey ______

Last name of person completing survey ______

Position ______

E-mail address ______

Phone number (+) ______

Please select the one item that best describes your organisation:

- ☐ NGO: hepatitis patient group
- ☐ NGO: direct service provider
- ☐ NGO: other (please describe: ______)
- ☐ Medical society
- ☐ Private foundation
- ☐ Other: ______

Annex B continued

Part B. Response to Information Reported by Governments

**Information reported by [country] government**<br>(All text in this column is copied from the Global Policy Report on the Prevention and Control of Viral Hepatitis in WHO Member States, 2013)	**Civil society perspective**<br>(please complete by selecting one check-box in each cell)
**1. National coordination**	
1.1. Government response to:<br>› In your country, is there a written national strategy or plan that focuses exclusively or primarily on the prevention and control of viral hepatitis?<br>› If yes, is it exclusive for viral hepatitis or does it also address other diseases? Please indicate components of the strategy or plan.	☐ To our knowledge, this information is accurate.<br>☐ To our knowledge, this information is not accurate.<br>☐ We take no position regarding this statement.<br>**Comments:** (200 words maximum)
1.2. Government response to:<br>› Is there a designated governmental unit/department responsible only for coordinating and/or carrying out viral hepatitis-related activities?<br>› If yes, what is its name?<br>› How many people work full-time (or how many full-time equivalent staff) on hepatitis-related activities in all government agencies/bodies?	☐ To our knowledge, this information is accurate.<br>☐ To our knowledge, this information is not accurate.<br>☐ We take no position regarding this statement.<br>**Comments:** (200 words maximum)
1.3. Government response to:<br>› Does your government have a viral hepatitis prevention and control programme that includes activities targeting specific populations?<br>› If yes, please indicate which populations.	☐ To our knowledge, this information is accurate.<br>☐ To our knowledge, this information is not accurate.<br>☐ We take no position regarding this statement.<br>**Comments:** (200 words maximum)
**2. Awareness-raising and partnerships**	
2.1. Government response to:<br>› Did your government hold events for World Hepatitis Day 2012?<br>› Has your government funded any public viral hepatitis awareness campaigns since January 2011, other than World Hepatitis Day?	☐ To our knowledge, this information is accurate.<br>☐ To our knowledge, this information is not accurate.<br>☐ We take no position regarding this statement.<br>**Comments:** (200 words maximum)
2.2. Government response to:<br>› Does your government collaborate with any civil society group within your country (such as patient groups or national or local nongovernmental organisations) to develop and implement its viral hepatitis prevention and control programme?<br>› If yes, please name major partners.	☐ To our knowledge, this information is accurate.<br>☐ To our knowledge, this information is not accurate.<br>☐ We take no position regarding this statement.<br>**Comments:** (200 words maximum)

Information reported by [country] government<br>(All text in this column is copied from the Global Policy Report on the Prevention and Control of Viral Hepatitis in WHO Member States, 2013)	Civil society perspective<br>(please complete by selecting one check-box in each cell)
**3. Evidence-based policy and data for action**	
3.1. Government response to:<br>› Is there routine surveillance for viral hepatitis?<br>› If yes, is there a national surveillance system for the following types of acute hepatitis? *A, B, C.*<br>› Is there a national surveillance system for the following types of chronic hepatitis? *B, C.*	☐ To our knowledge, this information is accurate.<br>☐ To our knowledge, this information is not accurate.<br>☐ We take no position regarding this statement.<br>**Comments:** (200 words maximum)
3.2. Government response to:<br>› Are there standard case definitions for hepatitis infections?<br>› Are deaths, including from hepatitis, reported to a central registry?<br>› What percentage of hepatitis cases are reported as "undifferentiated" or "unclassified" hepatitis?	☐ To our knowledge, this information is accurate.<br>☐ To our knowledge, this information is not accurate.<br>☐ We take no position regarding this statement.<br>**Comments:** (200 words maximum)
3.3. Government response to:<br>› Are liver cancer cases registered nationally?<br>› Are cases of HIV/hepatitis co-infection registered nationally?<br>› How often are hepatitis disease reports published?	☐ To our knowledge, this information is accurate.<br>☐ To our knowledge, this information is not accurate.<br>☐ We take no position regarding this statement.<br>**Comments:** (200 words maximum)
3.4. Government response to:<br>› Are hepatitis outbreaks required to be reported to the government?<br>› If yes, are they further investigated?<br>› Is there adequate laboratory capacity nationally to support viral hepatitis outbreak investigations and other surveillance activities?	☐ To our knowledge, this information is accurate.<br>☐ To our knowledge, this information is not accurate.<br>☐ We take no position regarding this statement.<br>**Comments:** (200 words maximum)
3.5. Government response to:<br>› Is there a national public health research agenda for viral hepatitis?<br>› Are viral hepatitis serosurveys conducted regularly?<br>› If yes, how often?<br>› When was the last one carried out? Please specify the target populations.	☐ To our knowledge, this information is accurate.<br>☐ To our knowledge, this information is not accurate.<br>☐ We take no position regarding this statement.<br>**Comments:** (200 words maximum)

Annex B continued

Information reported by [country] government<br>(All text in this column is copied from the Global Policy Report on the Prevention and Control of Viral Hepatitis in WHO Member States, 2013)	Civil society perspective<br>(please complete by selecting one check-box in each cell)
**4. Prevention of transmission**	
4.1. Government response to:<br>› Is there a national hepatitis A vaccination policy?<br>› If yes, what groups does the policy address?	☐ To our knowledge, this information is accurate.<br>☐ To our knowledge, this information is not accurate.<br>☐ We take no position regarding this statement.<br>**Comments:** (200 words maximum)
4.2. Government response to:<br>› Has your government established the goal of eliminating hepatitis B? If yes, in what timeframe?	☐ To our knowledge, this information is accurate.<br>☐ To our knowledge, this information is not accurate.<br>☐ We take no position regarding this statement.<br>**Comments:** (200 words maximum)
4.3. Government response to:<br>› Nationally, what percentage of newborn infants in a given recent year received the first dose of hepatitis B vaccine within 24 hours of birth?<br>› Nationally, what percentage of one-year-olds (ages 12–23 months) in a given recent year received three doses of hepatitis B vaccine?	☐ To our knowledge, this information is accurate.<br>☐ To our knowledge, this information is not accurate.<br>☐ We take no position regarding this statement.<br>**Comments:** (200 words maximum)
4.4. Government response to:<br>› Is there a national policy specifically targeting mother-to-child transmission of hepatitis B?	☐ To our knowledge, this information is accurate.<br>☐ To our knowledge, this information is not accurate.<br>☐ We take no position regarding this statement.<br>**Comments:** (200 words maximum)
4.5. Government response to:<br>› Is there a specific national strategy and/or policy/ guidelines for preventing hepatitis B and hepatitis C infection in health care settings?<br>› If yes, are health workers vaccinated against hepatitis B prior to starting work that might put them at risk of exposure to blood?	☐ To our knowledge, this information is accurate.<br>☐ To our knowledge, this information is not accurate.<br>☐ We take no position regarding this statement.<br>**Comments:** (200 words maximum)
4.6. Government response to:<br>› Is there a national policy on injection safety in health-care settings?<br>› If yes, what type of syringes does the policy recommend for therapeutic injections?<br>› Are single-use or auto-disable syringes, needles and cannulas always available in all health care facilities?	☐ To our knowledge, this information is accurate.<br>☐ To our knowledge, this information is not accurate.<br>☐ We take no position regarding this statement.<br>**Comments:** (200 words maximum)

**Information reported by [country] government**<br>(All text in this column is copied from the Global Policy Report on the Prevention and Control of Viral Hepatitis in WHO Member States, 2013)	**Civil society perspective**<br>(please complete by selecting one check-box in each cell)
4.7. Government response to:<br>› What are your government's official estimates of the number and percentage of unnecessary injections administered annually in health care settings? (e.g., injections that are given when an equivalent oral medication is available)	☐ To our knowledge, this information is accurate.<br>☐ To our knowledge, this information is not accurate.<br>☐ We take no position regarding this statement.<br>**Comments:** (200 words maximum)
4.8. Government response to:<br>› Is there a national infection control policy for blood banks?<br>› Are all donated blood units (including family donations) and blood products nationwide screened for hepatitis B?<br>› Are all donated blood units (including family donations) and blood products nationwide screened for hepatitis C?	☐ To our knowledge, this information is accurate.<br>☐ To our knowledge, this information is not accurate.<br>☐ We take no position regarding this statement.<br>**Comments:** (200 words maximum)
4.9. Government response to:<br>› Is there a national policy relating to the prevention of viral hepatitis among people who inject drugs?	☐ To our knowledge, this information is accurate.<br>☐ To our knowledge, this information is not accurate.<br>☐ We take no position regarding this statement.<br>**Comments:** (200 words maximum)
4.10. Government response to:<br>› Does your government have guidelines addressing how hepatitis A and hepatitis E can be prevented through food and water safety?	☐ To our knowledge, this information is accurate.<br>☐ To our knowledge, this information is not accurate.<br>☐ We take no position regarding this statement.<br>**Comments:** (200 words maximum)
**5. Screening, care and treatment**	
5.1. Government response to:<br>› How do health professionals in your country obtain the skills and competencies required to effectively care for people with viral hepatitis?<br>› Are there national clinical guidelines for the management of viral hepatitis?<br>› If yes, do they include recommendations for cases of HIV co-infection?<br>› If no, are there national clinical guidelines for the management of HIV that include recommendations for co-infection with viral hepatitis?	☐ To our knowledge, this information is accurate.<br>☐ To our knowledge, this information is not accurate.<br>☐ We take no position regarding this statement.<br>**Comments:** (200 words maximum)

Annex B continued

Information reported by [country] government<br>(All text in this column is copied from the Global Policy Report on the Prevention and Control of Viral Hepatitis in WHO Member States, 2013)	Civil society perspective<br>(please complete by selecting one check-box in each cell)
5.2. Government response to:<br>› Does your government have a national policy relating to screening and referral to care for hepatitis B? For hepatitis C?	☐ To our knowledge, this information is accurate.<br>☐ To our knowledge, this information is not accurate.<br>☐ We take no position regarding this statement.<br>**Comments:** (200 words maximum)
5.3. Government response to:<br>Please answer the following questions about hepatitis B and hepatitis C testing in your country.<br>› When testing, do people register by name?<br>› If people register by name, are their names kept confidential within the system, or is there open access to the names?<br>› Is the test free of charge for all individuals?<br>› Is the test free of charge for members of any specific group?<br>› Is the test compulsory for members of any specific group?	☐ To our knowledge, this information is accurate.<br>☐ To our knowledge, this information is not accurate.<br>☐ We take no position regarding this statement.<br>**Comments:** (200 words maximum)
5.4. Government response to:<br>› Is publicly funded treatment available for hepatitis B? If yes, who is eligible?<br>› Is publicly funded treatment available for hepatitis C? If yes, who is eligible?<br>› How much does the government spend on publicly funded treatment for hepatitis B and hepatitis C?	☐ To our knowledge, this information is accurate.<br>☐ To our knowledge, this information is not accurate.<br>☐ We take no position regarding this statement.<br>**Comments:** (200 words maximum)
5.5. Government response to:<br>› Which hepatitis B drugs and hepatitis C drugs are included on the national essential medicines list or are subsidised by the government?	☐ To our knowledge, this information is accurate.<br>☐ To our knowledge, this information is not accurate.<br>☐ We take no position regarding this statement.<br>**Comments:** (200 words maximum)

Part C. Key Hepatitis Policy Issues and Proposed Agenda for Change

For Part C, your organisation is asked to discuss the policy response to viral hepatitis in your country in greater depth, focusing on one or more of five topics listed below. Please follow these steps:

Choose one of the five topics:

1. National coordination
2. Awareness-raising, partnerships and resource mobilisation (WHO Axis 1)[1]
3. Evidence-based policy and data for action (WHO Axis 2)[1]
4. Prevention of transmission (WHO Axis 3)[1]
5. Screening, care and treatment (WHO Axis 4)[1]

Write your organisation's assessment of the national response to viral hepatitis as it relates to the topic you chose (maximum 400 words and please use the box below). Some points to consider are:

- What are the greatest problems with this component of the national response to viral hepatitis?
- What needs to change?
- What should be the government's role in bringing about these changes? What responsibilities should the government have?
- What should be the roles and responsibilities of other stakeholders at the community, national and international levels? (You may wish to list these in bullet points.)
- What evidence exists to support your organisation's viewpoint? (Consider, for example, citing surveys, research reports, statistics and newspaper articles.)

Please repeat these steps for as many of the five topics as you wish to address.

Topic 1

Topic 2

Topic 3

Topic 4

Topic 5

1. Prevention and control of viral hepatitis infection: framework for global action. Geneva, WHO, 2012 (http://who.int/csr/disease/hepatitis/Framework/en/index.html).

Annex B continued

World Hepatitis Alliance 2014 Survey of Civil Society Stakeholders

A response to the Global Policy Report on the Prevention and Control of Viral Hepatitis in WHO Member States (World Health Organization, 2013)

Your organisation's cooperation is requested in gathering data for the World Hepatitis Alliance's 2014 global hepatitis policy report. This report is seen as a civil society response to information provided by governments for the 2013 global hepatitis policy report published by the World Health Organization (http://www.who.int/csr/disease/hepatitis/global_report/en/).

All World Health Organization member states were surveyed for the 2013 report. Civil society organisations in countries where governments responded to the survey are being invited to comment on the information that their governments provided.

Your government **did not** respond to the 2013 survey. The World Hepatitis Alliance therefore would like to ask you to complete a modified version of its 2014 survey of civil society stakeholders. The following survey has two parts:

- In **Part A** your organisation is asked to provide basic information about itself.
- In **Part B** your organisation is asked to discuss the policy response to hepatitis in your country, focusing on issues that your organisation wishes to prioritise. Your organisation also is invited to put forth an "agenda for change" outlining proposed roles and responsibilities for key stakeholders.

Part A. Organisational information

Organisation name ____________________

Street address ____________________

City __________ Postal code/zip code __________

Country ____________________

Website ____________________

First name of person completing survey ____________________

Last name of person completing survey ____________________

Position ____________________

E-mail address ____________________

Phone number (+) ____________________

Please select the one item that best describes your organisation:

☐ NGO: hepatitis patient group

☐ NGO: direct service provider

☐ NGO: other (please describe: ____________________)

☐ Medical society

☐ Private foundation

☐ Other: ____________________

Part B. Key hepatitis policy issues and proposed agenda for change

For Part B, your organisation is asked to discuss the policy response to viral hepatitis in your country in greater depth, focusing on one or more of five topics listed below. Please follow these steps:

Choose one of the five topics:

1. National coordination
2. Awareness-raising, partnerships and resource mobilisation (WHO Axis 1)[1]
3. Evidence-based policy and data for action (WHO Axis 2)[1]
4. Prevention of transmission (WHO Axis 3)[1]
5. Screening, care and treatment (WHO Axis 4)[1]

Write your organisation's assessment of the national response to viral hepatitis as it relates to the topic you chose (maximum 400 words and please use the box below). Some points to consider are:

- What are the greatest problems with this component of the national response to viral hepatitis?
- What needs to change?
- What should be the government's role in bringing about these changes? What responsibilities should the government have?
- What should be the roles and responsibilities of other stakeholders at the community, national and international levels? (You may wish to list these in bullet points.)
- What evidence exists to support your organisation's viewpoint? (Consider, for example, citing surveys, research reports, statistics and newspaper articles.)

Please repeat these steps for as many of the five topics as you wish to address.

Topic 1

Topic 2

Topic 3

Topic 4

Topic 5

1. Prevention and control of viral hepatitis infection: framework for global action. Geneva, WHO, 2012 (http://who.int/csr/disease/hepatitis/Framework/en/index.html).
