# Supplementary material for: Strengthening hepatitis B and C surveillance in Europe: results from the two global hepatitis policy surveys (2013 and 2014)
Source: Hepatol Med Policy. 2016 Jun 30;1:3. doi: 10.1186/s41124-016-0009-5 (PMC5918699; doi:10.1186/s41124-016-0009-5)
Supplement: Supplementary file 3 — Reporting by Member States on the existence of a written national viral hepatitis strategy or plan. (DOCX 30 kb) [file 41124_2016_9_MOESM3_ESM.docx]

**Additional File 3. Reporting by Member States on the existence of a written national viral hepatitis strategy or plan**

|  | **World Health Organization European sub-region** | | | | | | **TOTAL** |
| --- | --- | --- | --- | --- | --- | --- | --- |
|  | **West** | | **Centre** | | **East** | |  |
|  | N=18 (%) | Countries | N=13 (%) | Countries | N=13 (%) | Countries | N=44 (%) |
| Existence of a written national strategy or plan that focuses exclusively or primarily on the prevention and control of viral hepatitis: | | | | | | | |
| *yes* | 5 (27.8) | Austria, Denmark, France, Israel, United Kingdom | 3 (23.1) | Czech Republic, Slovenia, Turkey | 5 (38.5) | Armenia, Kyrgyzstan, Moldova, Russian Federation, Uzbekistan | 13 (29.6) |
| *no* | 13 (78.2) | Andorra, Belgium, Finland, Germany, Ireland, Italy, Luxembourg, Malta, Netherlands, San Marino, Spain, Sweden, Switzerland | 10 (76.9) | Albania, Bulgaria, Croatia, Cyprus, Hungary, Montenegro, Poland, Serbia, Slovakia, The Former Yugoslav Republic of Macedonia | 8 (61.5) | Azerbaijan, Belarus, Estonia, Georgia, Latvia, Lithuania, Tajikistan, Ukraine | 31 (70.5) |
|  | | | | | | | |
| If there is a strategy or plan, does it include a surveillance component? | | | | | | | |
|  | N=5 (%) | Countries | N=3 (%) | Countries | N=5 (%) | Countries | N=13 (%) |
| *yes* | 5 (100) | Austria, Denmark, France, Israel, United Kingdom | 3 (100) | Czech Republic, Slovenia, Turkey | 4 (80.0) | Armenia, Kyrgyzstan, Moldova, Russian Federation | 12 (92.3) |
| *no* | 0 (0) |  | 0 (0) |  | 1 (20.0) | Uzbekistan | 1 (7.7) |
|  | | | | | | | |
| If there is a strategy or plan, is it exclusive for viral hepatitis or does it also address other diseases? | | | | | | | |
|  | N=5 (%) | Countries | N=3 (%) | Countries | N=5 (%) | Countries | N=13 (%) |
| *exclusive for viral hepatitis* | 1 (20.0) | France | 1 (33.3) | Czech Republic | 2 (40.0) | Kyrgyzstan, Moldova | 4 (30.8) |
| *only for HBV* | 0 (0) |  | 1 (33.3) | Turkey | 0 (0) |  | 1 (7.7) |
| *only for HCV* | 1 (20.0) | United Kingdom | 0 (0) |  | 0 (0) |  | 1 (7.7) |
| *integrated with other diseases* | 3 (60.0) | Austria, Denmark, Israel | 1 (33.3) | Slovenia | 3 (60.0) | Armenia, Russian Federation, Uzbekistan | 7 (53.8) |
