# Supplementary material for: Strengthening hepatitis B and C surveillance in Europe: results from the two global hepatitis policy surveys (2013 and 2014)
Source: Hepatol Med Policy. 2016 Jun 30;1:3. doi: 10.1186/s41124-016-0009-5 (PMC5918699; doi:10.1186/s41124-016-0009-5)
Supplement: Supplementary file 4 — Reporting by Member States on routine surveillance of viral hepatitis B and C. (DOCX 36 kb) [file 41124_2016_9_MOESM4_ESM.docx]

**Additional File 4. Reporting by Member States on routine surveillance of viral hepatitis B and C**

|  | **World Health Organization European sub-region** | | | | | | | **TOTAL** |
| --- | --- | --- | --- | --- | --- | --- | --- | --- |
|  | **West** | | | **Centre** | | **East** | |  |
|  | N=18 (%) | Countries | | N=13 (%) | Countries | N=13 (%) | Countries | N=44 (%) |
| National surveillance system for **acute HBV**: | | | | | | | | |
| *yes* | 17 (94.4) | Andorra, Austria, Belgium, Denmark, Finland, France, Germany, Ireland, Israel, Italy, Luxembourg, Malta, Netherlands, Spain, Sweden, Switzerland, United Kingdom | | 13 (100) | Albania, Bulgaria, Croatia, Cyprus, Czech Republic, Hungary, Montenegro, Poland, Serbia, Slovakia, Slovenia, The Former Yugoslav Republic of Macedonia, Turkey | 13 (100) | Armenia, Azerbaijan, Belarus, Estonia, Georgia, Kyrgyzstan, Latvia, Lithuania, Moldova, Russian Federation, Tajikistan, Ukraine, Uzbekistan | 43 (97.3) |
| *no* | 1 (5.6) | San Marino | | 0 (0) |  | 0 (0) |  | 1 (2.3) |
|  | | | | | | | | |
| National surveillance system for **acute HCV**: | | | | | | | | |
| *yes* | 15 (83.3) | Andorra, Austria, Belgium, Denmark, Germany, Ireland, Israel, Italy, Luxembourg, Malta, Netherlands, Spain, Sweden, Switzerland, United Kingdom | | 13 (100) | Albania, Bulgaria, Croatia, Cyprus, Czech Republic, Hungary, Montenegro, Poland, Serbia, Slovakia, Slovenia, The Former Yugoslav Republic of Macedonia, Turkey | 13 (100) | Armenia, Azerbaijan, Belarus, Estonia, Georgia, Kyrgyzstan, Latvia, Lithuania, Moldova, Russian Federation, Tajikistan, Ukraine, Uzbekistan | 41 (93.2) |
| *no* | 3 (16.7) | San Marino, Finland, France | | 0 (0) |  | 0 (0) |  | 3 (6.8) |
|  | | | | | | | | |
| National surveillance system for **chronic HBV**: | | | | | | | | |
| *yes* | 11 (61.1) | Austria, Denmark, Finland, France, Ireland, Luxembourg, Malta, Netherlands, Sweden, Switzerland, United Kingdom | | 7 (53.8) | Croatia, Cyprus, Montenegro, Poland, Serbia, Slovakia, Slovenia | 10 (76.9) | Belarus, Estonia, Georgia, Kyrgyzstan, Latvia, Lithuania, Moldova, Russian Federation, Tajikistan, Ukraine | 28 (63.6) |
| *no* | 7 (38.9) | Andorra, Belgium, Germany, Israel, Luxembourg, San Marino, Spain | | 6 (46.2) | Albania, Bulgaria, Czech Republic, Hungary, The Former Yugoslav Republic of Macedonia, Turkey | 3 (23.1) | Armenia, Azerbaijan, Uzbekistan | 16 (36.4) |
|  | | | | | | | | |
| National surveillance system for **chronic HCV**: | | | | | | | | |
| *yes* | 10 (55.6) | Austria, Denmark, Finland, France, Ireland, Luxembourg, Malta, Sweden, Switzerland, United Kingdom | | 7 (53.8) | Croatia, Cyprus, Montenegro, Poland, Serbia, Slovakia, Slovenia | 10 (76.9) | Belarus, Estonia, Georgia, Kyrgyzstan, Latvia, Lithuania, Moldova, Russian Federation, Tajikistan, Ukraine | 27 (61.4) |
| *no* | 8 (44.4) | Andorra, Belgium, Germany, Israel, Luxembourg, Netherlands, San Marino, Spain | | 6 (46.2) | Albania, Bulgaria, Czech Republic, Hungary, The Former Yugoslav Republic of Macedonia, Turkey | 3 (23.1) | Armenia, Azerbaijan, Uzbekistan | 17 (38.6) |
|  | | | | | | | | |
| Standard case definitions for viral hepatitis infections: | | | | | | | | |
| *yes* | 17 (94.4) | Andorra, Austria, Belgium, Denmark, Finland, France, Germany, Ireland, Israel, Italy, Luxembourg, Malta, Netherlands, Spain, Sweden, Switzerland, United Kingdom | | 13 (100) | Albania, Bulgaria, Croatia, Cyprus, Czech Republic, Hungary, Montenegro, Poland, Serbia, Slovakia, Slovenia, The Former Yugoslav Republic of Macedonia, Turkey | 12 (92.3) | Armenia, Azerbaijan, Belarus, Estonia, Georgia, Kyrgyzstan, Latvia, Lithuania, Moldova, Russian Federation, Tajikistan, Uzbekistan | 42 (95.5) |
| *no* | 1 (5.6) | San Marino | | 0 (0) |  | 1 (7.7) | Ukraine | 2 (4.5) |
|  | | | | | | | | |
| % of hepatitis cases reported as “undifferentiated” or “unclassified”: | | | | | | | | |
| *zero* | 6 (33.3) | Andorra, Ireland, San Marino, Sweden, Switzerland, United Kingdom | | 2 (15.4) | Cyprus, Slovenia | 2 (15.4) | Azerbaijan, Tajikistan | 10 (22.7) |
| *less than 5%* | 2 (11.1) | Israel, Netherlands | | 4 (30.8) | Croatia, Czech Republic, Poland, Serbia | 3 (23.1) | Armenia, Estonia, Latvia | 9 (20.5) |
| *5–15%* | 0 (0) |  | | 2 (15.4) | Bulgaria, The Former Yugoslav Republic of Macedonia | 3 (23.1) | Lithuania, Moldova, Russian Federation | 5 (11.4) |
| *more than 15%* | 1 (5.6) | Spain | | 2 (15.4) | Albania, Montenegro | 1 (7.1) | Kyrgyzstan | 4 (9.1) |
| *no response* | 9 (50.0) | Austria, Belgium, Denmark, Finland, France, Germany, Italy, Luxembourg, Malta | | 3 (23.1) | Hungary, Slovakia, Turkey | 4 (30.8) | Belarus, Georgia, Ukraine, Uzbekistan | 16 (36.4) |
|  | | | | | | | | |
| Adequate laboratory capacity nationally to support hepatitis outbreak investigations and other surveillance activities for HBV and HBC: | | | | | | | | |
| *yes* | 18 (100) | | Andorra, Austria, Belgium, Denmark, Finland, France, Germany, Ireland, Israel, Italy, Luxembourg, Malta, Netherlands, San Marino, Spain, Sweden, Switzerland, United Kingdom | 13 (100) | Albania, Bulgaria, Croatia, Cyprus, Czech Republic, Hungary, Montenegro, Poland, Serbia, Slovakia, Slovenia, The Former Yugoslav Republic of Macedonia, Turkey | 13 (100) | Armenia, Azerbaijan, Belarus, Estonia, Georgia, Kyrgyzstan, Latvia, Lithuania, Moldova, Russian Federation, Tajikistan, Ukraine, Uzbekistan | 44 (100) |
|  | | | | | | | | |
| Hepatitis outbreaks required to be reported to the government and further investigated: | | | | | | | | |
| *yes* | 17 (94.4) | Andorra, Austria, Belgium, Denmark, Finland, France, Germany, Ireland, Israel, Italy, Luxembourg, Malta, Netherlands, San Marino, Spain, Sweden, Switzerland | | 13 (100) | Albania, Bulgaria, Croatia, Cyprus, Czech Republic, Hungary, Montenegro, Poland, Serbia, Slovakia, Slovenia, The Former Yugoslav Republic of Macedonia, Turkey | 12 (92.3) | Armenia, Azerbaijan, Belarus, Estonia, Georgia, Kyrgyzstan, Latvia, Moldova, Russian Federation, Tajikistan, Ukraine, Uzbekistan | 42 (95.5) |
| *no* | 1 (5.6) | United Kingdom* | | 0 (0) |  | 1 (7.7) | Lithuania | 2 (4.5) |
|  | | | | | | | | |
| Hepatitis disease reports published regularly (at least once per year): | | | | | | | | |
| *yes* | 15 (83.3) | Andorra, Austria, Belgium, Denmark, Finland, France, Germany, Ireland, Israel, Italy, Netherlands, Spain, Sweden, Switzerland, United Kingdom | | 13 (100) | Albania, Bulgaria, Croatia, Cyprus, Czech Republic, Hungary, Montenegro, Poland, Serbia, Slovakia, Slovenia, The Former Yugoslav Republic of Macedonia, Turkey | 11 (84.6) | Armenia, Azerbaijan, Belarus, Estonia, Georgia, Kyrgyzstan, Latvia, Moldova, Russian Federation, Tajikistan, Uzbekistan | 39 (88.6) |
| *no* | 3 (16.7) | Luxembourg, Malta, San Marino | | 0 (0) |  | 2 (15.4) | Lithuania, Ukraine | 5 (11.4) |

* Outbreaks in Scotland, England, Wales and Northern Ireland are reported and investigated on corresponding regional level but not required to be reported nationally.
