# Supplementary material for: Strengthening hepatitis B and C surveillance in Europe: results from the two global hepatitis policy surveys (2013 and 2014)
Source: Hepatol Med Policy. 2016 Jun 30;1:3. doi: 10.1186/s41124-016-0009-5 (PMC5918699; doi:10.1186/s41124-016-0009-5)
Supplement: Supplementary file 5 — Reporting by Member States on other sources of strategic information and capacities for disease burden estimation. (DOCX 39 kb) [file 41124_2016_9_MOESM5_ESM.docx]

**Additional File 5. Reporting by Member States on other sources of strategic information and capacities for disease burden estimation**

|  | | **World Health Organization European sub-region** | | | | | | **TOTAL** |
| --- | --- | --- | --- | --- | --- | --- | --- | --- |
|  | | **West** | | **Centre** | | **East** | |  |
|  | | N=18 (%) | Countries | N=13 (%) | Countries | N=13 (%) | Countries | N=44 (%) |
| Deaths, including from hepatitis, reported to a central registry: | | | | | | | |  |
| *yes* | 17 (94.4) | Andorra, Austria, Belgium, Denmark, Finland, France, Germany, Ireland, Israel, Italy, Luxembourg, Malta, Netherlands, Spain, Sweden, Switzerland, United Kingdom | 13 (100) | Albania, Bulgaria, Croatia, Cyprus, Czech Republic, Hungary, Montenegro, Poland, Serbia, Slovakia, Slovenia, The Former Yugoslav Republic of Macedonia, Turkey | 12 (92.3) | Armenia, Azerbaijan, Belarus, Estonia, Georgia, Kyrgyzstan, Latvia, Lithuania, Moldova, Russian Federation, Tajikistan, Uzbekistan | 42 (95.5) |  |
| *no* | 1 (5.6) | San Marino | 0 (0) |  | 0 (0) |  | 1 (2.3) |  |
| *no response* | 0 (0) |  | 0 (0) |  | 1 (7.7) | Ukraine | 1 (2.3) |  |
|  | | | | | | | |  |
| Liver cancer cases registered nationally: | | | | | | | |  |
| *yes* | 15 (83.3) | Andorra, Austria, Belgium, Denmark, Finland, Germany, Ireland, Israel, Luxembourg, Malta, Netherlands, San Marino, Spain, Sweden, United Kingdom | 11 (84.6) | Bulgaria, Croatia, Cyprus, Czech Republic, Hungary, Poland, Serbia, Slovakia, Slovenia, The Former Yugoslav Republic of Macedonia, Turkey | 9 (69.2) | Armenia, Azerbaijan, Belarus, Latvia, Lithuania, Moldova, Russian Federation, Tajikistan, Ukraine | 35 (79.5) |  |
| *no* | 3 (16.7) | France, Italy, Switzerland | 2 (15.4) | Albania, Montenegro | 3 (23.1) | Estonia, Georgia, Kyrgyzstan | 8 (18.2) |  |
| *do not know* | 0 (0) |  | 0 (0) |  | 1 (7.7) | Uzbekistan | 1 (2.3) |  |
|  | | | | | | | |  |
| Cases of HIV/hepatitis co-infection registered nationally: | | | | | | | |  |
| *yes* | 10 (55.6) | *Andorra, Austria, Belgium, Denmark, Finland, France, Luxembourg, Netherlands, San Marino, Spain* | 10 (76.9) | *Albania, Bulgaria, Croatia, Cyprus, Czech Republic, Montenegro, Serbia, Slovakia, Slovenia, The Former Yugoslav Republic of Macedonia* | 9 (69.2) | *Armenia, Azerbaijan, Belarus, Estonia, Georgia, Kyrgyzstan, Latvia, Russian Federation, Tajikistan* | 29 (65.9) |  |
| *no* | 8 (44.4) | *Germany, Ireland, Israel, Italy, Malta, Sweden, Switzerland, United Kingdom* | 3 (23.1) | *Hungary, Poland, Turkey* | 4 (30.8) | *Lithuania, Moldova, Ukraine, Uzbekistan* | 15 (34.1) |  |
|  | | | | | | | |  |
| Viral hepatitis serosurveys conducted regularly: | | | | | | | |  |
| *yes* | 8 (44.4) | Belgium, Denmark, Finland, France, Germany, Netherlands, Sweden United Kingdom | 5 (38.5) | Albania, Bulgaria, Montenegro, Serbia, Slovenia | 7 (53.8) | Armenia, Azerbaijan, Belarus, Moldova, Russian Federation, Tajikistan, Ukraine | 20 (45.5) |  |
| *no* | 10 (55.6) | Andorra, Austria, Ireland, Israel, Italy, Luxembourg, Malta, San Marino, Spain, Switzerland | 6 (46.2) | Croatia, Czech Republic, Hungary, Poland, Former Yugoslav Republic of Macedonia, Turkey | 6 (46.2) | Estonia, Georgia, Kyrgyzstan, Latvia, Lithuania, Uzbekistan | 22 (50.0) |  |
| *do not know* | 0 (0) |  | 2 (15.4) | Cyprus, Slovakia | 0 (0) |  | 2 (4.5) |  |
| Target populations for serosurveys (respondents were given the options of “children”, “general population”, “people who inject drugs”, “men who have sex with men”, and “other groups”, with space for identifying other groups). | | | | | | | |  |
| *children* | 2 (11.1) | Germany, Sweden | 1 (7.7) | Croatia | 2 (15.4) | Moldova, Russian Federation | 5 (11.4) |  |
| *general population* | 4 (22.2) | Belgium, France, Germany, Netherlands | 5 (38.5) | Albania, Bulgaria, Croatia, Czech Republic, Slovenia | 2 (15.4) | Moldova, Russian Federation | 11 (25.0) |  |
| *people who inject drugs* | 4 (22.2) | Denmark, Finland, France, United Kingdom | 3 (23.1) | Croatia, Montenegro, Slovenia | 5 (38.5) | Belarus, Moldova, Russian Federation, Tajikistan, Ukraine | 12 (27.3) |  |
| *men who have sex with men* | 2 (11.1) | Finland, France | 4 (30.8) | Albania, Croatia, Montenegro, Serbia | 3 (23.1) | Belarus, Tajikistan, Ukraine | 9 (20.5) |  |
| *pregnant women* | 2 (11.1) | Denmark, Finland | 2 (15.4) | Albania, Slovenia | 5 (38.5) | Armenia, Azerbaijan, Belarus, Latvia, Tajikistan | 9 (20.5) |  |
| *prisoners* | 2 (11.1) | France, Netherlands | 2 (15.4) | Croatia, Montenegro | 2 (15.4) | Belarus, Tajikistan | 6 (13.6) |  |
| *health care workers* | 0 (0) |  | 2 (15.4) | Albania, Slovenia | 4 (30.8) | Azerbaijan, Armenia, Belarus, Russian Federation | 6 (13.6) |  |
| *sex workers* | 1 (5.6) | Finland | 2 (15.4) | Montenegro, Serbia | 2 (15.4) | Tajikistan, Ukraine | 5 (11.4) |  |
| *people living with HIV* | 0 (0) |  | 1 (7.7) | Slovenia | 2 (15.4) | Belarus, Tajikistan | 3 (6.8) |  |
| *blood donors* | 0 (0) |  | 1 (7.7) | Slovenia | 3 (23.1) | Armenia, Azerbaijan, Moldova | 4 (9.1) |  |
| *military* | 0 (0) |  | 0 (0) |  | 2 (15.4) | Belarus, Latvia | 2 (4.5) |  |
| *Roma youth* | 0 (0) |  | 1 (7.7) | Serbia | 0 (0) |  | 1 (2.3) |  |
|  | | | | | | | |  |
| All pregnant women screened for HBV: | | | | | | | |  |
| *yes* | 16 (88.9) | Andorra, Austria, Belgium, Denmark, Finland, France, Germany, Ireland, Italy, Luxembourg, Netherlands, San Marino, Spain, Sweden, Switzerland, United Kingdom | 9 (69.2) | Croatia, Cyprus, Hungary, Montenegro, Poland, Serbia, Slovakia, Slovenia, Turkey | 9 (69.2) | Armenia, Azerbaijan, Belarus, Estonia, Georgia, Latvia, Moldova, Russian Federation, Ukraine | 34 (77.3) |  |
| *no* | 2 (11.1) | Israel, Malta | 4 (30.8) | Albania, Bulgaria, Czech Republic, The Former Yugoslav Republic of Macedonia | 4 (30.8) | Kyrgyzstan, Lithuania, Tajikistan, Uzbekistan | 10 (22.7) |  |
|  | | | | | | | |  |
| All donated blood (including family donations) screened for HBV: | | | | | | | |  |
| *Yes* | 17 (94.4) | Austria, Belgium, Denmark, Finland, France, Germany, Ireland, Israel, Italy, Luxembourg, Malta, Netherlands, San Marino, Spain, Sweden, Switzerland, United Kingdom | 13 (100) | Albania, Bulgaria, Croatia, Cyprus, Czech Republic, Hungary, Montenegro, Poland, Serbia, Slovakia, Slovenia, The Former Yugoslav Republic of Macedonia, Turkey | 12 (100) | Armenia, Azerbaijan, Belarus, Estonia, Georgia, Latvia, Lithuania, Moldova, Russian Federation, Tajikistan, Ukraine, Uzbekistan | 42 (95.5) |  |
| no | 0 (0) |  | 0 (0) |  | 1 (7.7) | Kyrgyzstan | 1 (2.3) |  |
| *N/A (no blood centres)* | 1 (5.6) | Andorra | 0 (0) |  | 0 (0) |  | 1 (2.3) |  |
|  | | | | | | | |  |
| All donated blood (including family donations) screened for HCV: | | | | | | | |  |
| *yes* | 17 (94.4) | Austria, Belgium, Denmark, Finland, France, Germany, Ireland, Israel, Italy, Luxembourg, Malta, Netherlands, San Marino, Spain, Sweden, Switzerland, United Kingdom | 13 (100) | Albania, Bulgaria, Croatia, Cyprus, Czech Republic, Hungary, Montenegro, Poland, Serbia, Slovakia, Slovenia, The Former Yugoslav Republic of Macedonia, Turkey | 13 (100) | Armenia, Azerbaijan, Belarus, Estonia, Georgia, Latvia, Lithuania, Moldova, Russian Federation, Tajikistan, Ukraine, Uzbekistan | 43 (97.7) |  |
| *N/A (no blood centres)* | 1 (5.6) | Andorra | 0 (0) |  | 0 (0) |  | 1 (2.3) |  |
