# Supplementary material for: Strengthening hepatitis B and C surveillance in Europe: results from the two global hepatitis policy surveys (2013 and 2014)
Source: Hepatol Med Policy. 2016 Jun 30;1:3. doi: 10.1186/s41124-016-0009-5 (PMC5918699; doi:10.1186/s41124-016-0009-5)
Supplement: Supplementary file 6 — Reporting by Member States regarding areas in which they might want assistance from WHO. (DOCX 25 kb) [file 41124_2016_9_MOESM6_ESM.docx]

**Additional File 6. Reporting by Member States regarding areas in which they might want assistance from WHO**

|  | **World Health Organization European sub-region** | | | | | | **TOTAL** |
| --- | --- | --- | --- | --- | --- | --- | --- |
|  | **West** | | **Centre** | | **East** | |  |
|  | N=18 (%) | Countries | N=13 (%) | Countries | N=13 (%) | Countries | N=44 (%) |
| Areas in which government might want WHO assistance: | | | | | | | |
| *national plan* | 2 (11.1) | Belgium, San Marino | 5 (38.5) | Bulgaria, Cyprus, Montenegro, Slovakia, The Former Yugoslav Republic of Macedonia | 10 (76.9) | Armenia, Azerbaijan, Estonia, Georgia, Kyrgyzstan, Latvia, Moldova, Russian Federation, Tajikistan, Ukraine | 17 (38.6) |
| *surveillance* | 1 (5.6) | Belgium | 1 (7.7) | Montenegro | 8 (61.5) | Armenia, Azerbaijan, Georgia, Kyrgyzstan, Latvia, Moldova, Russian Federation, Tajikistan | 10 (22.7) |
| *burden estimation* | 1 (5.6) | Belgium | 6 (46.2) | Albania, Croatia, Montenegro, Poland, Serbia, Slovenia | 8 (61.5) | Georgia, Kyrgyzstan, Latvia, Lithuania, Moldova, Russian Federation, Tajikistan, Ukraine | 15 (34.1) |
